# Engineering Nanoparticle Surface Amphiphilicity: An Integrated Computational and Laser Desorption Ionization Study of Controlled Ligand Self-Assembly

**DOI:** 10.1021/acs.jpcc.5c03644

**Published:** 2025-08-13

**Authors:** Jacob Kennedy, Zachary LaFaver, Marcus Dupart, Kateri H. DuBay, David L. Green

**Affiliations:** † Department of Chemical Engineering, 2358 University of Virginia, Charlottesville, Virginia 22903, United States; ‡ Department of Chemistry, 2358 University of Virginia, Charlottesville, Virginia 22903, United States; § Department of Materials Science and Engineering, 2358 University of Virginia, Charlottesville, Virginia 22903, United States

## Abstract

Multiligand monolayers can self-organize into advantageous interfacial patterns that govern nanoparticle (NP) properties. Polyethylene glycol (PEG) is widely incorporated into self-assembled monolayers to enhance biocompatibility, particularly in drug delivery applications. Previous studies demonstrate that monolayer phase separation can be controlled by leveraging the energetic and entropic driving forces acting on ligands in the design of amphiphilic surfaces. In this work, we extend an integrated experimental and simulation framework to investigate the self-assembly of dodecanethiol (DDT), a long hydrophobic alkanethiol, with 2-ethoxyethane-1-thiol, a short hydrophilic PEG-thiol, as a function of their surface composition on ultrasmall gold NPs. The PEG-DDT Au NPs were synthesized via ligand exchange. Integrated MALDI-MS experiments and configurationally biased Monte Carlo simulations were used to analyze and predict the local ordering of the surface ligands. The MALDI-MS fragment distributions obtained from experiment and simulation show quantitative agreement, and both indicate that the PEG-DDT ligands undergo phase separation, resulting in NP monolayers with patchy to Janus-like hydrophilic and hydrophobic ligand domains. Further, the domain size was found to increase proportionally with the surface fraction of each ligand, thereby demonstrating the ability to tune patch sizes in amphiphilic monolayers by controlling the surface composition.

## Graphical abstract

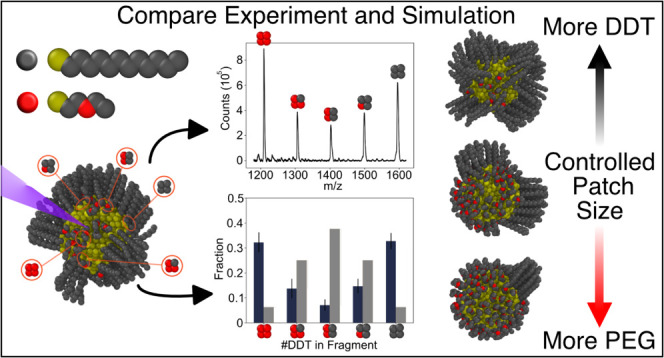

## Introduction

Monolayer-protected nanoparticles (NPs) have remarkably versatile and highly tunable properties, including protein adsorption modulation, NP stability, and therapeutic activity.^1–4^ These properties are largely governed by the adsorbed self-assembled monolayer (SAM), which can be engineered to incorporate multiple ligand-types, resulting in mixed monolayer shells that alter functionality and enhance performance. Amphiphilic monolayers, with both hydrophobic and hydrophilic domains, can promote highly specific interactions with blood serum, improve systemic (bio)­distribution, and enhance the targeting of cells and tissues.^5^ To this end, strategic ligand selection enables the development of NP-based drugs with tailorable surfaces.^6–10^ Moreover, the adsorbed ligand mixtures are translationally mobile on most nanocarrier interfaces.^11^ Consequently, the ligands can self-assemble into characteristic monolayer patterns that could, in theory, be tuned to optimize targeting and delivery. However, predicting and controlling monolayer pattern formation – a missing piece – presents a significant and open research challenge.

Controlling monolayer self-assembly is essential and ubiquitous to the formulation of diagnostic and therapeutic nanocarriers (e.g., proteins, viruses, micelles, vesicles, liposomes, dendrimers, coacervates, inorganic NPs, etc.). Often, hydrophilic polyethylene glycol (PEG) chains are incorporated into the attached monolayer to mitigate clearance by the immune system, thereby imparting “stealth” properties.^12–14^ In addition, the “PEGylated” interface on nanocarriers may include a second, chemically distinct ligand, often a lipid that better coordinates with the hydrophobic regions of proteins. The benefit is the improvement of in vivo biological response.^14^ Studies have consistently shown that a high ligand density of PEG within the mixed monolayer is a requirement for therapeutic effectiveness.^14^ Since NP function and SAM structure are inextricably linked, SAM structure characterization is the starting point for the development of structure–property-function relationships for the enhancement of PEGylated NP drugs. To date, their effective application has been hampered by a lack of analytical methods having the appropriate performance to accurately capture monolayer structure and its impact on NP interactions within complex biological systems.^15–18^

Current techniques to experimentally characterize surface morphology are limited. Scanning tunnel microscopy (STM) is capable of providing high-resolution, angstrom-level images that can be used to discern surface patterns in SAMs, however the technique is highly technical and interpretation is difficult for STM images on highly curved, ultrasmall NPs less than 10 nm in diameter, as noted by Lévy and Moriarty.^17^ Other techniques include transmission electron microscopy (TEM),^19,20^ small-angle neutron scattering (SANS),^21–24^ NMR,^25^ FTIR,^26^ and UV–vis,^27^ all of which can be used to measure properties related to the NP cores and can provide statistical information about the organization of their monolayers.^28,29^ Unfortunately, these techniques are limited by the high cost of materials, access to high-energy facilities, complex sample preparation, and resolution issues. Matrix-assisted laser desorption ionization mass spectrometry (MALDI-MS) provides an alternative and more accessible analysis technique that greatly simplifies sample preparation, while reducing material costs and instrumentation needs.^6,24,30–34^ We and others have shown that the laser ablation of functionalized gold and silver NPs results in intact, metal–ligand fragments with characteristic ratios that represent small, nanoscale pieces of the NP-SAM interface.^6,24,31–33^ Thus, while MALDI-MS provides a clear measure of ligand clustering in the monolayer, an atomistic-scale picture of the NP monolayer cannot be obtained from MALDI-MS alone. Recently, some have leveraged reverse Monte Carlo (RMC) calculations to generate monolayer morphologies that are consistent with the experimental data from SANS^23^ and MALDI-MS^24^ spectra to facilitate their interpretation. However, the structural information in RMC comes from simply rearranging 2D ligand surface patterns on the NP surface to match experimental data, rather than from models that include the actual physical forces acting upon three-dimensional ligands. As a result, structural information about the ligands themselves are not accessible with this approach, which limits potential extensions of the RMC techniques to monolayer prediction and design.

Numerical simulations that include physical forces acting between ligands can provide critical information regarding the forces that drive their self-organization, the atomistic monolayer structures, and how they change with different ligand combinations. Early work utilizing Dissipative Particle Dynamics (DPD) employed simplified NP shapes and interaction potentials and provided a framework for understanding the interplay between the entropic and enthalpic driving forces for ligand mixing.^35^ However, the simplified potentials employed in those efforts do not enable atomistic-level information or predictions for specific ligand combinations.^23,36–38^ More detailed potentials have been successfully employed in full MD simulations of homoligand monolayers;^39^ though, the much longer time scales required for the equilibration of mixed ligand monolayers (up to 4 days in the lab^40^) are not accessible via MD.^41^ One way to overcome the time scale limitations is to employ Monte Carlo (MC) algorithms with unphysical moves that enable the more rapid equilibration of the ligand monolayer. Configurationally Biased Monte Carlo (CBMC), which includes unphysical moves in the MC moveset such as ligand swaps, was designed to rapidly equilibrate oligomer and polymeric systems.^42,43^

In our early work, we demonstrated the power and ease of combining MALDI-MS analysis with CBMC simulations that included realistic representations of the physical driving forces in these NP-ligand systems. We found that the resulting metal–ligand MALDI-MS fragment distributions can be predicted by CBMC for a range of binary ligand mixtures and surface fractions, enabling the visualization of the monolayers of alkanethiols and mercaptoalcohols on silver NPs.^6,31^ Validated by a careful, quantitative, comparison to experimental MALDI-MS data, this CBMC approach provides complementary atomic-scale details from the equilibrated ligand structures, such as patch sizes and cocrystallization patterns.

Building upon the unified experimental-theoretical framework we have developed, our goal in this work is to extend these tools to determine the monolayer structure of PEGylated ultrasmall gold NPs. In this work, we quantify the patch sizes in amphiphilic binary monolayers composed of dodecanethiol (DDT), a 12-carbon hydrophobic alkanethiol, combined with 2-ethoxyethane-1-thiol, a short hydrophilic PEG-thiol. By synthesizing gold NPs with varying PEG surface fraction, we quantify how the ligand shell composition influences monolayer arrangement. Using experimental NP core sizes and interpolated surface ligand densities, CBMC simulations were performed to obtain equilibrated 3D structures of PEG-DDT Au NPs of varying PEG-DDT ratios. The ligand distributions predicted from CBMC agreed well with the MALDI-MS measurements of the real NPs. Thus, the simulation-derived morphologies can serve as atomically resolved representations of the NP monolayers, enabling further analyses. By tuning the composition of the SAM, we ultimately seek to control the hydrophilic and hydrophobic domains – a crucial step toward improving biodistribution and drug delivery.^44–46^

## Materials and Methods

### Experiments

#### Nanoparticle Synthesis

##### Reagents and Materials

Hydrogen tetrachloroaurate­(III) hydrate (99.99% trace metals purity), sodium borohydride (99.99% trace metals purity), 1-dodecanethiol (DDT, ≥ 98% purity), and trans-2-[3­(4-*tert*-butylphenyl)-2-methyl-2-propenylidene]­malonitrile (DCTB, ≥ 99% purity) were purchased from Sigma-Aldrich. 2-ethoxyethane-1-thiol (≥98% purity) was purchased from Enamine. Ethanol (200 proof, anhydrous) was sourced from Decon Laboratories. Ultrathin (<3 nm) carbon film on 400 mesh copper holey carbon grids for TEM imaging were purchased from Ted Pella. A Sorvall Biofuge Stratos Centrifuge from Kendro Laboratories was used for cleaning the nanoparticles.

##### Synthesis of PEG-DDT Au NPs

The mixed-ligand protected, PEG-DDT Au NPs were synthesized via a scalable one-step reduction method^47^ combined with a subsequent ligand-exchange step. First, hydrogen tetrachloroaurate­(III)­hydrate (*HAuCl*_4_) underwent reduction by sodium borohydride (*NaBH*_4_) in the presence of 1-dodecanethiol (DDT). The concentrations of *HAuCl*_4_, *NaBH*_4_, and DDT were 4.5 mM, and the reaction proceeded at room temperature. The rapid reduction of Au^3+^ to Au^0^ nucleated Au NPs with a diameter of roughly 2 – 4 nm in roughly 100 ms.^48^ Subsequently, DDT adsorbed onto the nuclei, halting NP growth,^40^ whereby the ligand acted as a capping agent, forming a monolayer on the NP surface. The reaction proceeded for 1 h to ensure monolayer formation and the synthesis of uniform, single monolayer, DDT Au NPs. The resulting suspension was then subjected to a three-step centrifugation washing process at 12,000 rpm for 15 min, with stepwise resuspension of the DDT Au NPs in ethanol to remove excess ligands and other impurities. A ligand exchange reaction was then performed to synthesize the PEG-DDT Au NPs by introducing excess quantities of the PEG ligand, 2-ethoxyethane-1-thiol, to a suspension of DDT-Au NPs. Prior to ligand exchange, the dry mass of DDT-capped NPs was quantified by vacuum drying the pellet for 72 h followed by subsequent weighing. The diameter of the synthesized NPs was measured via STEM, and their volume and surface area were then calculated accordingly. eq 1 was used to determine the volume of added PEG, *V*_PEG_, where *m*_dry_ is the dry mass of the vacuumed DDT Au NP sample, *A*_NP_ the NP surface area; ρ_DDT_, and ρ_PEG_ represent the bulk densities of DDT and PEG; *R* is the desired excess ratio of added PEG to attached DDT; *M*_PEG_ and *M*_DDT_ are respectively the PEG and DDT molecular weights, the DDT molecular weight; *V*_NP_ the NP volume, and *N*_A_ the Avogadro’s number.

$$V_{\mathrm{PEG}}=\frac{m_{\mathrm{dry}}A_{\mathrm{NP}}\rho_{\mathrm{DDT}}^{2}RM_{\mathrm{PEG}}}{\left[\frac{A_{\mathrm{NP}}\rho_{\mathrm{DDT}}M_{\mathrm{DDT}}}{N_{A}}+V_{\mathrm{NP}}\rho_{\mathrm{Au}}\right]N_{A}\rho_{\mathrm{PEG}}}$$ 1

The resultant volume of PEG was added to the DDT-protected Au NP sample and stirred to facilitate the ligand exchange reaction. This reaction proceeded for 72 h to ensure the mixed monolayer reaches equilibrium.

#### Nanoparticle Characterization

The successful syntheses of the single-monolayer DDT Au NPs and the mixed-ligand PEG-DDT Au NPs were determined by the following methods. UV–vis spectroscopy provided qualitative confirmation of a narrow Au core size and distribution through detection of the Au surface plasmon resonance (SPR). Subsequently, the analyses of transmission electron microscopy (TEM) and scanning transmission electron microscopy (STEM) images were used determine the average Au core diameter and size distribution (*d*_c_ ± Δ*d*_c_). MALDI-MS was used to determine the experimental nearest-neighbor ligand distribution of the PEG-DDT Au NPs for comparison to the theoretical CBMC simulations.

##### UV–Vis Spectroscopy

All UV–vis measurements of the NP samples were taken on a Shimadzu UV-2450/2550 spectrophotometer. The suspending NP solvent, ethanol, was used as a blanking agent. The NP concentration for each sample was diluted to obtain absorbance readings within the single particle scattering limit. The measured resonant UV–vis energy spectrum – which in principle varies with NP size, shape, and core material – provides information regarding NP formation and ligand attachment. In particular, Au NPs exhibit a SPR absorbance peak around λ = 510–580 nm, with smaller Au NPs exhibiting an SPR near 510 nm.^49^ The presence of an alkanethiol monolayer has also been shown to increase (i.e., redshift) the SPR of Au NPs.^50^ This range is used as a quality control check for successful NP synthesis and sufficient cleaning of washed NP solutions. An SPR near λ = 540 nm was found for the NP samples as shown in Figure S2 in the Supporting Information.

##### Electron Microscopy

The average NP size and distribution were determined using TEM and STEM. TEM micrographs were captured with a FEI Titan 80–300 TEM at an accelerating voltage of 300 kV. The same voltage was used during the collection of STEM micrographs on Thermo Fisher Themis instrument. TEM grid sample preparation was performed via the drop mounting method: a pipet was used to place a drop of NP solution on the corner of the TEM grid, and a small piece of paper underneath the grid was used to wick away extra solvent. The grids were dried at 50 °C for 30 min prior to analysis. The average NP core size and distribution (*d*_c_ ± Δ*d*_c_) were determined through image analysis with ImageJ software.^51^

##### Mass Spectrometry

MALDI-MS measurements were performed on a Shimadzu 8030 time-of-flight (TOF) instrument with a 337 nm nitrogen laser operating at 200 Hz. Samples for measurement were prepared by first dissolving 25 mg of the matrix-assist agent DCTB in 1 mL of methanol, followed by the addition via pipet of 2 μL of a solution containing 2 mg/mL PET-DDT NPs in ethanol. Subsequently, 1 μL of each resultant matrix-sample solution was spotted in the sample wells of a stainless steel, 48-well, Fleximass-SR48 MALDI-MS sample plate. Immediately following sample deposition, the MALDI-MS plate was placed under vacuum to remove solvent and facilitate crystallization of the matrix-NP sample. Data collection per sample was optimized at a laser power that varied between 25 and 35 (out of 100) within the settings of the instrument. Its time-of-flight detector was operated in linear, positive ion mode, with a pulsed extracted mass of 2465 Da and ion gate blanking of 700 Da. The instrument has an inherent resolution of 5000 fwhm and was calibrated to accuracy within 500 mDa prior to each run.

Laser ablation disintegrates the PEG-DDT Au NPs, resulting in characteristic, intact gold-ligand fragments that report on monolayer self-assembly. Hence, the fragments provide an opportunity to stochastically sample the binary mixed-ligand monolayer, whereupon the further analysis of a MALDI-MS spectrum can yield the nearest-neighbor ligand distribution. The process starts by detecting the relative abundances of the characteristic gold–thiolate complexes that contain the two ligand-types through their mass-to-charge ratios. They confer the ability to identify the number of each ligand-type on a metal fragment through knowledge of their respective molecular weights. Functionalized Au NPs typically yield characteristic fragments of the following generic formula: Au_*n*_ *L_n_*, where *n* is an integer (typically between 2 and 8), tracking the total number of ligands (*L*) on a fragment in tandem with the same number of Au atoms. The parameter *L* represents the combination of the two ligand-types A and B on a fragment as follows: Au_*n*_A_*i*_B_*n*–*i*_, where the integer index *i* = 0···*n*.^32–34,41,52^ The most predominant species is typically the Au_4_L_4_ fragment, which comprises a distinct family of five metal–ligand fragments based on the following ways that A and B can combine on an Au_4_ fragment: A_0_B_4_, A_1_B_3_, A_2_B_2_, A_3_B_1_, and A_4_B_0_, wherein the subscripts represent the number of A and B for a total of *n* = 4 ligands on a fragment. In this manuscript, ligand-types A and B are respectively, DDT and PEG.

Harkness et al. demonstrated that normalization of a MALDI-MS spectrum results in a metal–ligand fragment distribution, corresponding to the nearest-neighbor ligand distribution.^33,34,52^ The analysis of which yields the relative (molar) surface fractions of the ligands within the mixed-monolayer and establishes scoring functions that provide quantitative measures of phase separation for comparison to theory. As such, the experimental ligand distribution (θ_*i*,exp_) is obtained by calculating the area under each fragment-peak, *C*_*i*_, through integration, normalized by the total area under all peaks (eq 2). The surface fraction of ligand-type A, *x*_*A*_, is calculated by weighting each fragment-distribution peak, θ_*i*,exp_, by the corresponding number of A ligands, *A*_*i*_, on that fragment, normalized by the total number of ligands, *n*, on a fragment (eq 3). Accordingly, the surface fraction of ligand-type B, *x*_*B*_ = 1 – *x*_*A*_, is obtained through a mole balance.

$$\theta_{i,\exp}=\frac{C_{i}}{\overset{n}{\underset{i=0}{\sum }}C_{i}}$$ 2

$$x_{A}=\frac{\overset{n}{\underset{i=0}{\sum }}A_{i}\theta_{i,\exp}}{n}$$ 3

The sum-of-squares residual (SSR), computed via eq 5, provides a measure of the deviation from random ligand mixing through comparison of the experimental ligand distribution to a binomial distribution in eq 4. The latter predicts a randomly distributed mixed-ligand monolayer based on the surface fractions, *x*_*A*_ and *x*_*B*_, and number of ways the ligand-types A and B can combine on a fragment. Consequently, if A and B are randomly distributed, each potential combination of the two ligand-types on a fragment represents a Bernoulli trial. Hence, every fragment family constitutes a series of Bernoulli trials. Within the Au_4_A_i_B_4‑i_ fragment-family, the Au_4_A_2_B_2_ fragment has the highest anticipated probability corresponding to a randomly mixed SAM.^31^ As such, a normalized experimental MALDI-MS spectrum of random SAM-NPs yields low SSR ≈ 0.001, which gradually increase in value (SSR ≈ 0.3–0.6) over two-orders of magnitude with higher degrees of phase separation as the ligand shell evolves toward a patchy or Janus morphology.^6,30,47^

θi,binom=(ni)xAi(1−xA)n−i 4

$$\mathrm{SSR}=\sum_{i=0}^{n}(\theta_{i}-\theta_{i,\mathrm{binom}}{)}^{2}$$ 5

Eqs 2–5, encapsulating the experimental SSR and nearest-neighbor ligand distribution from a MALDI-MS spectrum, provide quantitative measures of monolayer phase separation for direct comparison to simulation, as detailed below.

### Simulation

The atomistic simulations were based on a structurally accurate, 3D-model of PEG-DDT Au NPs. Its core size was chosen based on the sizes of the experimental nanoparticles, as obtained from STEM data (see Figure 2). The adsorbed ligand densities were also chosen to match previously observed experimental values for ligand monolayers of PEG-related thiols^53^ and DDT.^54^ Configurationally biased Monte Carlo (CBMC)^6,31,43,55^ was then used to compute equilibrium monolayer morphologies, enabling the prediction of the MALDI-MS ligand distributions as well as their corresponding SSR values, as compared to a random distribution of ligands.

The specific 3D Au-SAM NP model used in this study was developed previously and has been used successfully to investigate the dependence of ligand length, chemical mismatch, and surface composition for a variety of alkanethiol and mercapto-alcohol SAMs on Ag NPs.^6,31^ Here, we extend the simulations to model ultrasmall Au cores decorated with a mixture of 2-ethoxyethane-1-thiol, a thiol-PEG, and DDT, a longer alkanethiol. The Au core was realistically modeled as a 20-facet Mackay icosahedron^56^ with a mid-diameter of 2.8 nm, matching the experimental size from TEM/STEM (see Figure 2). The Au atoms within the simulated core were arranged in a face-centered cubic (FCC) lattice with a lattice constant of 4.08 Å, which corresponds to the experimentally measured Au lattice constant.^57,58^ To reduce the computational load, only the outer three layers of the Au core were included in the simulations. The core was fixed and centered at the origin of the simulation box.

The PEG and DDT ligands in the simulations are represented by the Optimized Potentials for Liquid Simulations united atom (OPLS-UA) model,^59–61^ wherein only the hydrogen atoms bonded to noncarbon atoms are explicitly modeled. Thus, there are no hydrogen atoms included in the simulations presented here. The united atom DDT ligand is represented by 13 particles, one for each carbon in the chain plus the additional sulfur anchor. The 2-ethoxyethane-1-thiol ligand is represented by 6 particles, one for the sulfur, four for the carbons, and one for the ether oxygen.

The binding between the ligands’ anchoring sulfur atoms and gold surface atoms were modeled via a Morse potential.^39,62–64^ Harmonic bonds, angles, and OPLS dihedrals were employed to describe the intraligand interactions. Nonbonded, interligand interactions were modeled with a 12–6 Lennard-Jones potential with a cutoff of 10 Å and the Debye-screened Coulombic potential with a cutoff of 12 Å. To approximate the experimental conditions of ethanol – the suspending solvent for the experimental PEG-DDT Au NPs – an inverse Debye length of 5 Å and a dielectric constant of 24.35 ^65^ were employed. We used LAMMPS^66^ to evaluate the energetics, while the MC transformations were performed in Python with coordinate updates made to the LAMMPS instance through the LAMMPS Python module, subject to the randomly chosen move and acceptance.

Since the phase separation is sensitive to the surface density of the ligands (*L*) on the nanoparticle, we aimed to perform the simulations at full monolayer coverage, which correspond to the experimental conditions.^47^ To achieve the correspondence, the weighted-average of the known alkyl packing density on gold,^54^ 4.7 L/nm^2^, and an interpolated PEG surface density based on chain length,^53^ 6.29 L/nm^2^, was used to estimate the ligand interfacial density (σ) within the monolayer as a function of the PEG surface fraction (*x*_PEG_). For the simulations, *x*_PEG_ was varied from 0.1 to 0.9. The σ and *x*_PEG_ were used to determine the number of adsorbed PEG and DDT ligands, respectively *L*_PEG_ and *L*_DDT_, employed at each *x*_PEG_ in the simulations. The resulting surface compositions are detailed in Table 1 below.

**Table 1:** Simulated Number of PEG and DDT Ligands (*L*_PEG_ and *L*_DDT_) for Each *x*_PEG_ Value Were Computed from a Weighted-Average Ligand Density (σ) and *x*_PEG_ ^a^

*x*_PEG_	σ (L/nm^2^)	*L*_PEG_	*L*_DDT_	*L*_total_
0.1	4.86	12	111	123
0.2	5.02	25	102	127
0.3	5.18	39	92	131
0.4	5.34	54	82	136
0.5	5.50	70	70	140
0.6	5.66	87	58	145
0.7	5.73	105	45	150
0.8	5.84	123	30	153
0.9	6.04	143	15	158

a A total of 18 trials, initialized from different morphologies, were performed for every *x*_PEG_.

The CBMC algorithm speeds up the sampling of ligand conformations by enabling the proposal of larger moves while avoiding physically unrealistic chain collisions, thereby greatly increasing the acceptance rates of trial moves. This increased sampling efficiency becomes more important with the dense monolayers found in the experimental systems, as close packing causes most of the configurational space to be highly energetically unfavorable. Hence, CBMC simulations were used to predict the equilibrium monolayer morphology of the simulated PEG-DDT Au NP over the compositions listed in Table 1. Our CBMC move set consists of five different moves chosen probabilistically: Translation, Rotation, Regrowth, Swap, and Jump. Translation moves displace a randomly chosen ligand along the surface up to a maximum of 4 Å. The Rotation move rotates a randomly chosen ligand about a randomly chosen monomer in the chain up to a maximum of 0.1745 radians. Both the Translation and Rotation moves are accepted according to the standard Metropolis acceptance criterion^67^ and are each 10× more likely to be proposed than the latter three moves. The configurationally biased Regrowth move follows the chain regrowth algorithm presented by Siepmann and Frenkel,^55^ whereby a ligand is regrown in place using the Rosenbluth weight which gives higher preference to more energetically favorable chain configurations. The regrowth process involves reducing a randomly selected ligand down to the sulfur and three monomer beads to maintain the initial dihedral angle. Subsequently, the chain is regrown monomer by monomer with each regrown monomer sampling five orientations around the preceding monomer in the chain. The position relative to the preceding monomer in the chain is chosen probabilistically based on the relative energetics of each sampled orientation. The Regrowth move is then accepted or rejected according to the ratio of the Rosenbluth weights (*W*_trial_/*W*_initial_) which maintains detailed balance enabling equilibrium calculations.^55^ The Swap and Jump moves both involve a regrowth step and are therefore also configurationally biased and accepted according to the same criterion. The Swap move swaps the positions of two unlike ligands selected at random and then both ligands undergo regrowth. The Jump move randomly selects a ligand, which then randomly samples 20 available gap sites on Au facets within 10 – 20 Å, followed by ligand regrowth at the newly chosen surface site.

All simulations were run at 298.15 K. To determine whether the simulations reached equilibrium, a total of 18 trials were conducted for each *x*_PEG_ in Table 1. Based on the methodology used in our previous publications,^6,31^ the 18 trials for every *x*_PEG_ were divided into three smaller subgroups of six simulations, each initialized from unique configurations that fall into the following three different monolayer categories: Random, Striped, and Janus. Subsequently, we primarily used the following two checks to conclude with a high degree of confidence that an equilibrium state had been reached. First, the energy evolution of each trial plateaued indicating that a stationary distribution had been achieved. The trials were then allowed to sample for a sufficiently ample period of additional MC moves postplateau. Second, the low variation of SSR_sim_ values across the 18 trials for each *x*_PEG_ indicates a good convergence and equilibration from the three different types of starting configurations (see Figure 1). The final 10 configurations, spaced by 10,000 MC proposals each, were considered in the analysis of each trial. Further, because the SSR value depends on *x*_PEG_, we computed the SSR from 50 randomly designed, perfectly Janus, unrelaxed configurations at each surface fraction to determine the upper SSR bound at each *x*_PEG_, which is represented by the boundary of the shaded region in Figure 1.

**Figure 1:**
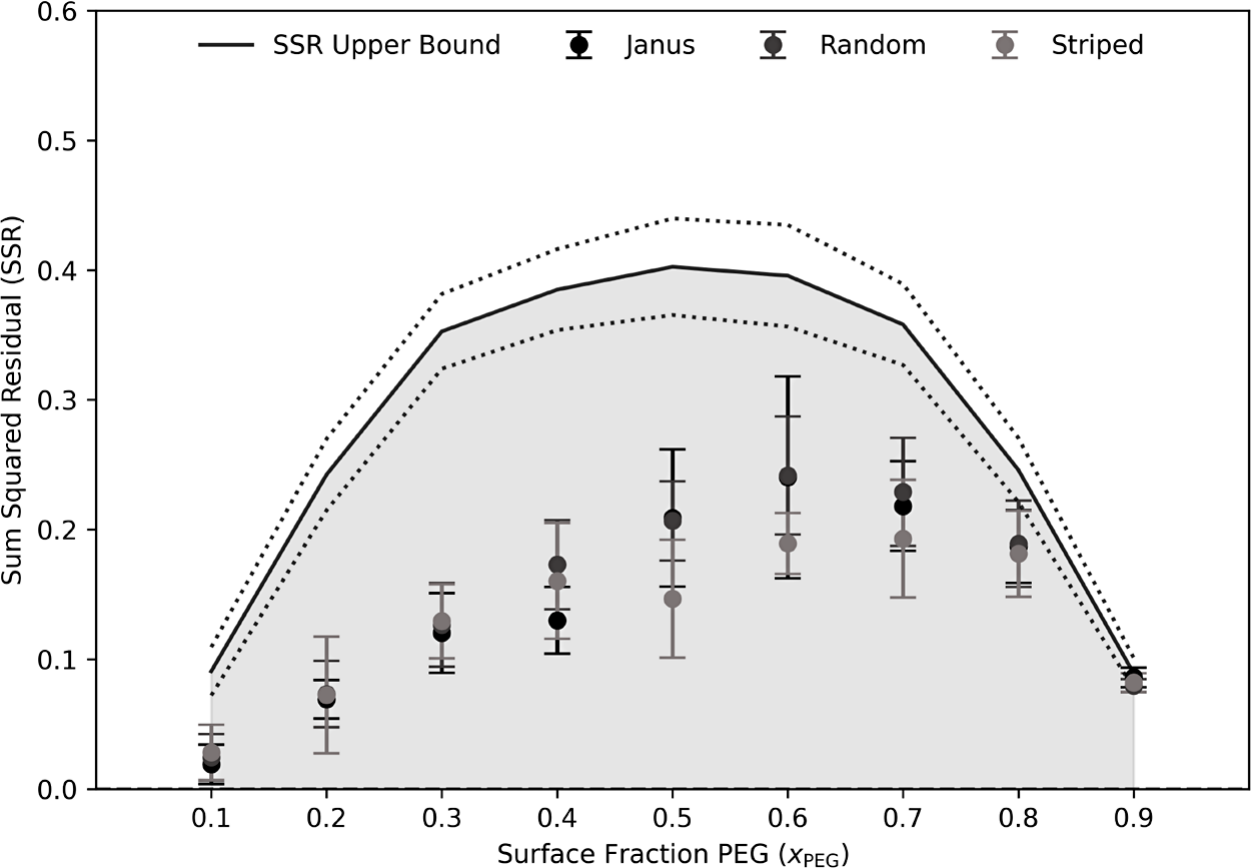
SSR values for all three initial configuration data sets (6 trials initially Janus, Random, and Striped) for each surface fraction (*x*_PEG_). The markers report the average of 60 configurations from the 6 trials in each subset all initialized in the corresponding morphology and the error bars report the standard deviation of those 60 configurations’ approximated fragment distributions. The boundary of the shaded region represents the average SSR value of 50 random, perfectly Janus, morphologies at each surface fraction and thus represents the upper bound for each *x*_PEG_ composition. The dotted lines above and below the solid line represent one standard deviation. SSR convergence is observed across all PEG surface fractions examined which suggests that the simulated morphologies have all equilibrated.

To build simulated MALDI-MS distributions from the MC calculations, we sample 4-ligand fragments from the surface of the equilibrated model PEG-DDT Au NP, since the Au_4_L_4_ fragments are produced in highest concentration during laser ablation. First, one ligand is randomly selected. Then the identities of its three nearest-neighbors (within 6 Å) are obtained to construct a 4-ligand fragment. Finally, this random sampling process is repeated 50,000 times for each of the equilibrated monolayer structures, thereby capturing the statistics of local ligand arrangement in the model SAM. A histogram is then constructed by sorting the collected fragments into the following categories: PEG_0_DDT_4_, PEG_1_DDT_3_, PEG_2_DDT_2_, PEG_3_DDT_1_, and PEG_4_DDT_0_, where the subscripts denote the number of PEG and DDT on a fragment (see Figure 3).

We compare the heights of this histogram (θ_*i*,exp_) for *i* = 0,1,2,3,4, which represents the number of DDT ligands in the fragment directly to the normalized MALDI-MS spectrum peak heights (θ_*i*,exp_) from the experiments (see eq 2). The simulated and experimental ligand distributions, along with the peaks of the corresponding random binomial distribution (θ_*i*,binom_) in eq 4, were substituted into eq 5 to determine the resulting SSR_sim_ and SSR_exp_ for quantitative comparison. The simulated and experimental ligand distributions and their corresponding SSR values, as compared to the binomial distribution, can be seen in Figures 4 and 5, respectively.

To determine the patch sizes of PEG and DDT from the equilibrated MC simulations, we implemented a depth-first search (DFS) algorithm with a KDTree that facilitated the efficient look-up of the nearest neighbors within a cutoff distance of 6 Å and ensured each ligand was only considered once during the patch size analysis. This search and sort method enabled the determination of the number of patches present in a given mixed-ligand SAM configuration and the number of ligands in each patch. Only the distance between the anchor sulfur atoms of neighboring ligands and the final 10 configurations of each trial were considered during the patch size analysis. The findings are presented in Figure 7.

## Results and Discussion

We begin with a discussion of the PEG-DDT Au NP system, focusing on its components, synthesis, and characterization via STEM and MALDI-MS. Then we discuss the computational 3D NP model and the process to compare the simulated MALDI-MS ligand distributions and SSR values to the experimental data. From both approaches of experiment and simulation, we obtain distributions of 4-ligand fragment clusters and discuss the information they provide regarding the degree of phase separation in the NP monolayer. Finally, we make use of the simulated atomistic structures to shed light on the morphology of the PEG-DDT monolayers in greater detail.

### Synthesis and Characterization of PEG-DDT Au NPs

As detailed in Materials and Methods, ultrasmall gold nanoparticles were synthesized with mixed monolayers of dodecanethiol (DDT) and 2-ethoxyethane-1-thiol (PEG) ligands. We chose to pair DDT with PEG as PEG is commonly used to modify (or PEGylate) the surfaces of nanocarriers for drug delivery. Moreover, these two ligand types present a good test system, as the interactions between the more polar PEG ligand and the nonpolar DDT ligand are expected to drive microphase separation in the NP monolayer, leading to patchy or Janus-like structures.

The ratio of PEG to DDT in the synthesis solution was varied to control the ligand ratio on the NP surface. Figure 2 shows representative shape (Figure 2a) and size distributions (Figure 2b) of the gold NPs synthesized with the PEG-DDT mixed monolayers. The Au NP cores in Figure 2a are roughly spherical, ranging from 1.0 to 4.5 nm in diameter with a median diameter and standard deviation of 2.8 ± 0.3 nm, as viewed in Figure 2b.

**Figure 2:**
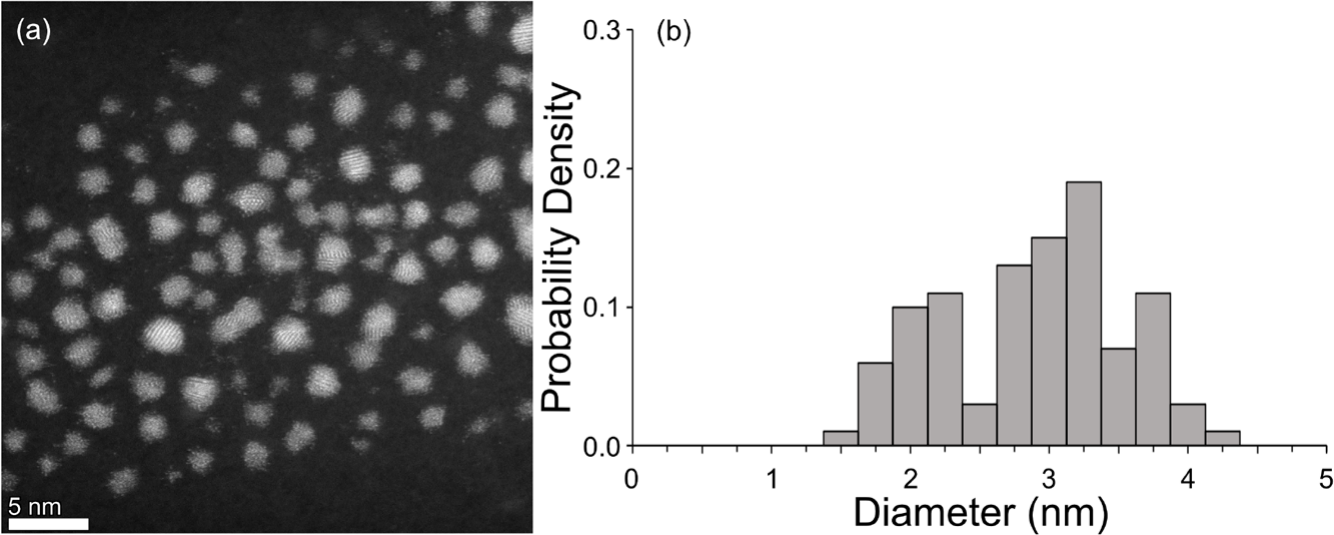
NP shape and size distribution from STEM. (a) Representative STEM image of gold NPs with dodecanethiol (DDT) and 2-ethoxyethane-1-thiol (PEG) monolayer. (b) Histogram of the particle sizes derived from STEM measurements. For a total of 100 counted particles, the median diameter is 2.8 nm with a standard deviation of 0.3 nm.

### Experimental and Simulated MALDI-MS Results

The synthesized NPs were analyzed via MALDI-MS to quantify the degree of phase separation in the PEG-DDT monolayer as a function of the ratio of PEG and DDT on the surface of each NP sample. At the same time, MALDI-MS was used to confirm that the experimental PEG surface fraction varied across the full range from *x*_PEG_ = 0.10 – 0.90. The analysis of the MALDI-MS spectra, as detailed in the methods section, focused on the Au_4_L_4_ fragment family due to the greater abundance of these fragments (and highest signal-to-noise ratio) relative to the other fragment families produced during laser ablation. With two distinguishable ligand-types, the Au_4_L_4_ fragment family exhibits five distinct MS peaks that correspond to the following ligand combinations: 4 PEG/0 DDT, 3 PEG/1 DDT, 2 PEG/2 DDT, 1 PEG/3 DDT, and 0 PEG/4 DDT. The left of Figure 3 (i.e., Figure 3a) displays an experimental MALDI-MS spectrum of these peaks for a surface fraction of PEG (*x*_PEG_) equal to 0.5. If the ligands are randomly mixed within the monolayer, the Au_4_L_4_ peaks will follow a binomial distribution. Thus, deviation from a binomial distribution represents deviation from a randomly mixed monolayer. In Figure 3c, both the binomial distribution (gray) and the experimentally determined ligand distribution (navy) are shown. We quantify the deviation from the binomial distribution by taking the sum of the squared residuals (SSR_exp_) of each of the experimental peaks from the expected binomial distribution at the measured value of *x*_PEG_, as represented by the relation to the right of Figure 3c. Thus, the SSR quantifies the nonrandom nature of the NP monolayer. Generally, as the SSR value increases, the degree of phase separation among the ligands composing the monolayer increases.

**Figure 3:**
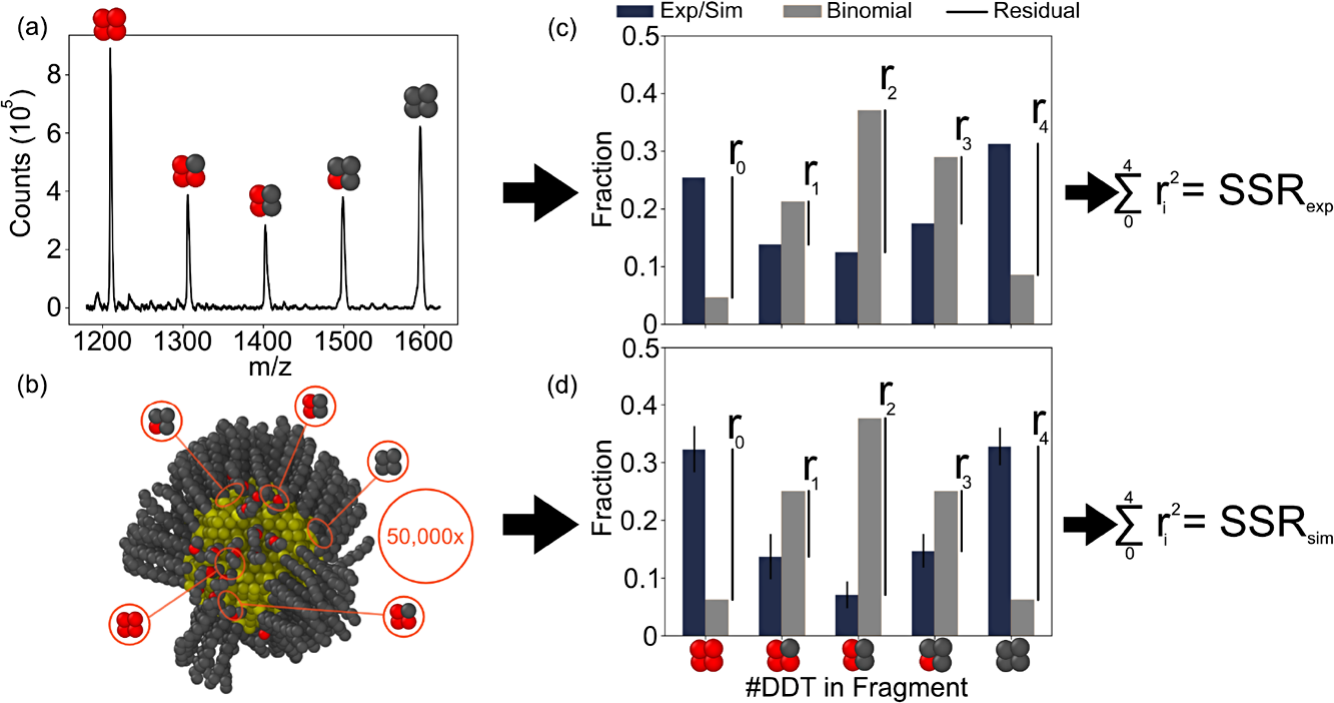
Schematic showing how experiment and simulation are compared via MALDI-MS fragment distributions. (a) Representative raw MALDI-MS spectrum of a PEG-DDT Au NP with of *x*_PEG_ = 0.47. Au_4_L_4_ fragment peaks are represented by 4 beads, one for each ligand, with gray beads representing DDT and red beads representing PEG. (b) Illustration of method to extract expected MALDI-MS distributions from the atomistic simulations. Here, an equilibrated snapshot of the corresponding *x*_PEG_ = 0.50 is shown. Ovito^68^ was used to visualize the snapshots obtained from simulation. The carbon in the ligands is shown in dark gray, sulfur in yellow, oxygen in red, and the Au NP core is gold. To build up the expected distributions, the equilibrated monolayer is randomly sampled 50,000× for simulated 4-ligand fragment clusters (circled in red). Each cluster is then classified by the number of DDT, resulting in a normalized fragment distribution (d) which can be then directly compared to experiment (c). The distributions of the experimental (c) and computational (d) results are subsequently compared to the binomial distribution for the corresponding ligand surface ratio. A quantitative comparison is then made by comparing the experimental and simulation SSR values. Adapted from reference 31. Copyright 2018 American Chemical Society.

The data obtained from the MALDI-MS experiments are then compared in Figures 4 and 5 to the predictions from the CBMC simulations run at the nine values of *x*_PEG_ ranging from 0.10 to 0.90. The process for developing the 3D model of the PEG-DDT Au NP and the algorithm to determine the expected MALDI-MS spectrum (see Figure 3b,d) are discussed in the Materials and Methods section. From the simulated and experimental MALDI distributions in Figure 4, we calculated both the SSR_exp_ and SSR_sim_ values, which are plotted in Figure 5, to quantify the degree of phase separation observed experimentally and via simulation.

**Figure 4:**
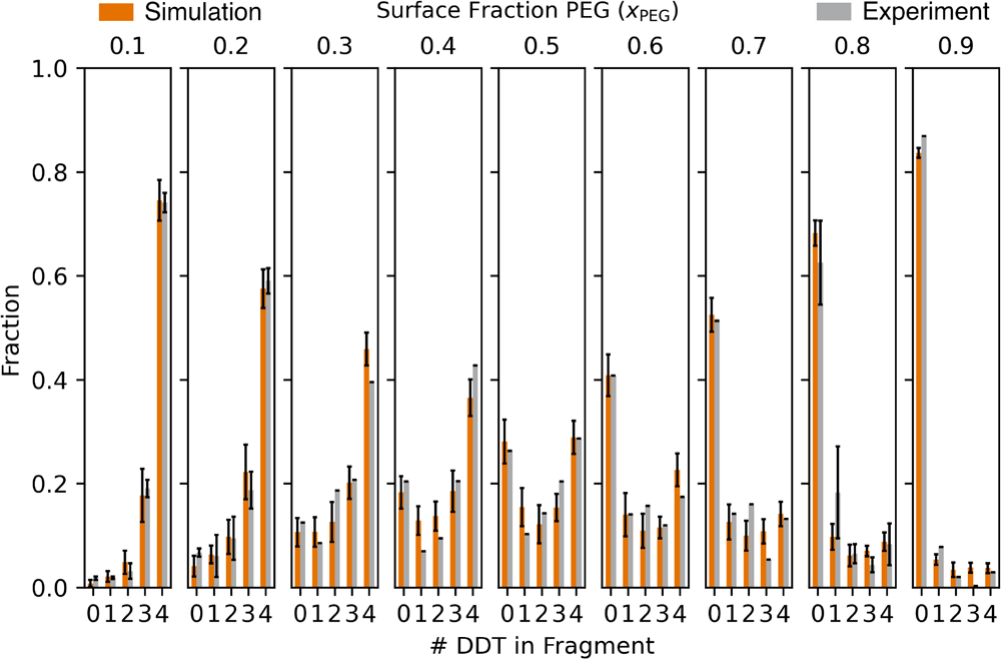
Comparisons of the normalized MALDI-MS ligand distributions between the closest experimental distribution (gray) to the average simulated distribution (orange) for the PEG-DDT Au NPs. Nine surface fractions were investigated, ranging from *x*_PEG_ = 0.10 – 0.90. The closest experimental distribution was chosen for comparison unless multiple experimental distributions fell within ± 0.015 *x*_PEG_ in which case they were averaged. The averaged distributions included *x*_PEG_^sim^ = 0.1, 0.2, and 0.8 with 3, 4, and 2 contributing experimental fragment distributions, respectively. Each experimentally averaged distribution is shown with standard deviation represented in the error bars (black). For simulation, the bar heights report the average of the final 10 configurations for each trial (180 total fragment distributions) and the error bars represent the standard deviation observed among the 180 configurations. The SSR_exp‑sim_ values for each *x*_PEG_, which report on the agreement between experiment and simulation, are listed in SI Table S1.

**Figure 5:**
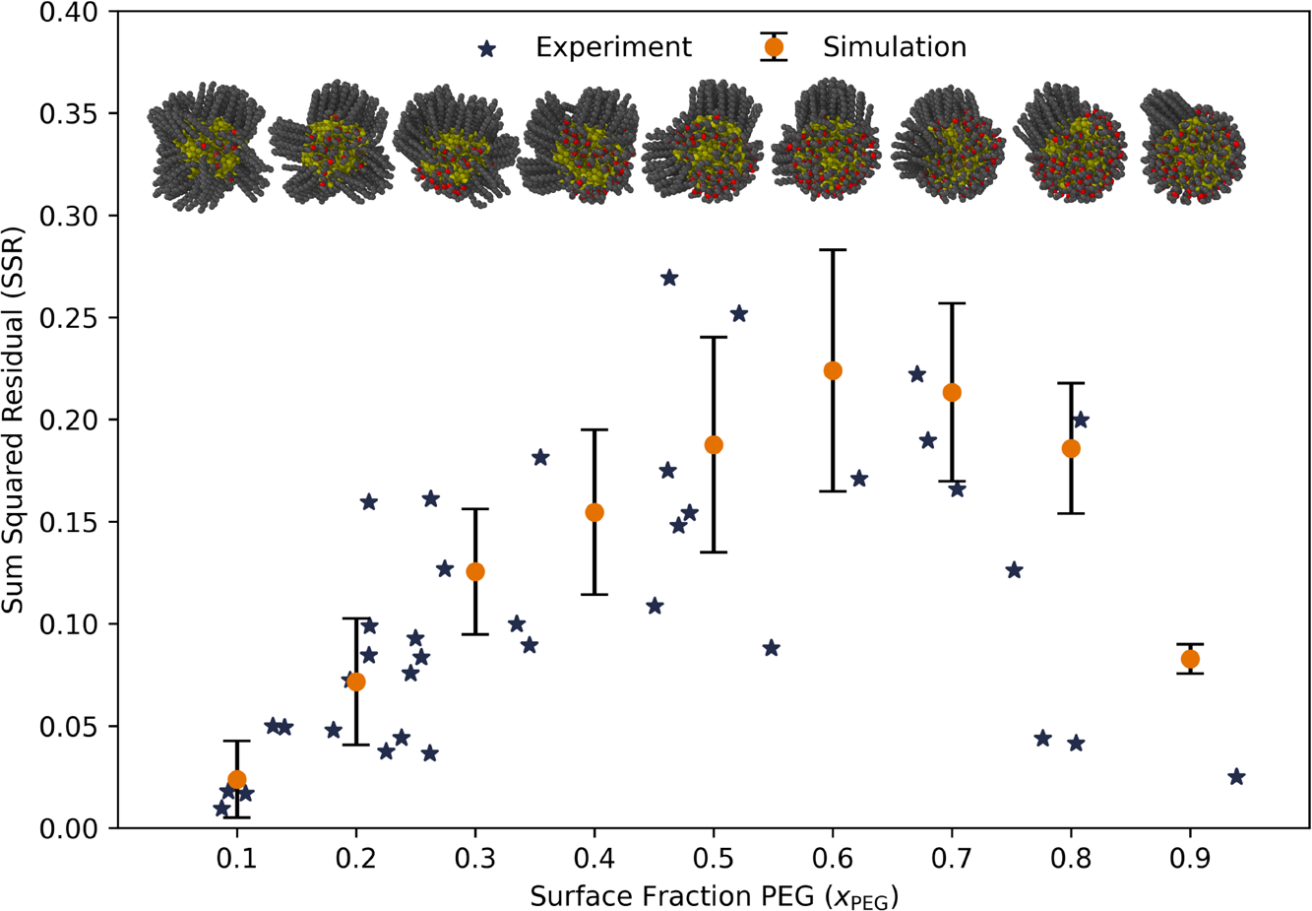
Experimental (navy stars) and simulation (orange circles) SSR values as a function of PEG surface fraction. While the experimental results are spread continuously across the range of *x*_PEG_ (the experimental determination of *x*_PEG_ is described in the Methods section), the simulations were conducted at discrete PEG surface fractions ranging from 0.1 to 0.9. Eighteen trials were run for each surface fraction with a third initialized in a Janus morphology, another third in a random morphology, and the remaining third in a striped morphology (stripes were 5 Å wide). The orange circle markers represent the simulation average SSR obtained from the 18 trials for each surface fraction data set and the error bars report the standard deviation. Representative snapshots, chosen randomly, from simulation are shown above the corresponding PEG surface fraction for added clarity. (See SI Figure S1 for individual simulation results at each *x*_PEG_.).

To quantitatively interrelate theory and experiment, the experimentally and computationally generated spectra are plotted in Figure 4 across the experimentally determined and computationally input PEG surface fractions, *x*_PEG_. Moreover, the comparisons of SSR_sim_ and SSR_exp_ are shown in Figure 5 as a function of *x*_PEG_. The simulation MALDI-MS distribution and its standard deviation error bars in Figure 4 are composed of 18 simulations for every *x*_PEG_. We compare each one to the experimental distribution with the closest PEG surface fraction. When multiple experimental MALDI-MS distributions were within 1.5% of a simulated *x*_PEG_ (3 for *x*_PEG_ = 0.10, 4 for *x*_PEG_ = 0.20, and 2 for *x*_PEG_ = 0.80), the average experimental distribution was calculated and standard deviations are shown with error bars.

The comparisons in Figure 4 show excellent matches between the experimental MALDI-MS distributions (gray) and the expected MALDI-MS distributions produced from the CBMC simulations (orange). For *x*_PEG_ = 0.40 – 0.60, larger peaks can be seen in the experimental and simulated mass distributions for both the 0DDT/4PEG and 4DDT/0PEG fragments while the peaks for the mixed ligand fragments in between are lower, consistent with a highly patchy or Janus-like monolayer. At *x*_PEG_ values below 0.4 and above 0.6, both mass distributions skew toward the ligand at higher concentration.

As outlined in Figure 3, the SSR values in Figure 5 were obtained by taking the sum of the squared residuals between the binomial distribution for the given *x*_PEG_ value and its respective experimental and simulated MALDI-MS distributions. The values of SSR_exp_ (navy stars) and SSR_sim_ (orange circles) are then plotted against *x*_PEG_. The SSR values from our simulations match well to the experimental data. The spread in SSR values is in line with our previous work on highly phase-separated dodecanethiol (DDT)/mercaptoethanol (ME) monolayer-protected silver NPs, where we demonstrated that the variance in SS*R*_exp_ could be increased by the presence of smaller diameter NPs within the DDT-ME Ag NP size distribution.^31^ Hence, the spread in SSR_exp_ in this work is likely to stem from the fraction of PEG-DDT Au NPs below the median core diameter of 2.8 nm, based on Figure 2b.

Additionally, the SSR value is dependent on *x*_PEG_, which must be considered to interpret the morphology from this number. This dependence is shown with shading in Figure 1, where the SSR values of 50 random, perfectly Janus, unrelaxed configurations at each *x*_PEG_ were calculated to obtain an upper bound on this number across *x*_PEG_. The SSR values become increasingly sensitive to minor variations in the monolayer organization, even in perfectly Janus structures at values of *x*_PEG_ close to 0.5 (see dotted lines in Figure 1). More significant reductions in SSR values, as compared to the perfectly Janus upper bound, are expected for patchy structures or Janus-like structures with boundaries influenced by the underlying icosahedral NP structure. For all surface fractions, however, we see that the rough metric of an SSR value greater than 0.1 should indicate a large degree of phase separation.^6,52,69^ We quantify the Janus-like character and the patch sizes below in Figures 6 and 7.

**Figure 6:**
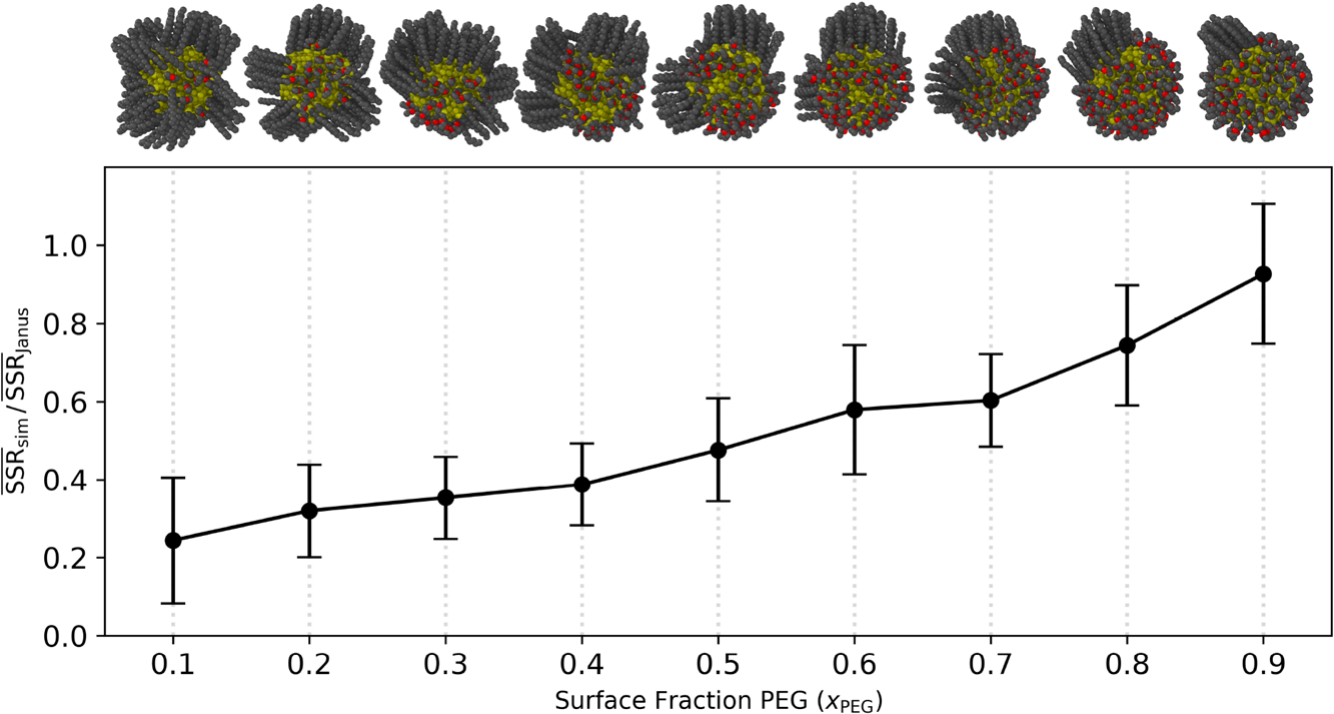
Ratio of the average SSR_sim_ and the average SSR_Janus_ as a function of *x*_PEG_. This metric reports on the degree of Janus character by comparing the observed SSR_sim_ values to that of a perfectly Janus counterpart, SSR_Janus_. The standard deviation is propagated from the quotient of the two averages and presented in the error bars, and the solid line was added to aid viewing. Representative snapshots were obtained from the equilibrated CBMC simulations and are included above the plot in order of increasing *x*_PEG_.

We also computed another quantitative measure, SSR_exp‑sim_, to report more directly on the agreement between the experimental and computational MALDI-MS distributions by calculating the SSR value between these two distributions directly. The SSR_exp‑sim_ values calculated from the distributions in Figure 4 were all found to be less than 0.03, indicating impressive agreement between the simulated and experimental MALDI-MS distributions across all *x*_PEG_ (see Table S1 in SI). Similar agreement was found in our previous studies that employed this methodology on a variety of mixed-ligand silver NP monolayers.^6,31^

### Analysis of Simulated PEG-DDT Au NP Monolayer Morphologies

While the SSR values in Figure 5 offer a quantitative metric for assessing monolayer phase separation, the metric lacks detailed structural information about ligand arrangement. Further insights underlying ligand phase separation can be drawn by analyzing the atomistic monolayer structures obtained from our simulations. Figure 6 displays the trend in Janus-like character, computed as the quotient of the average SSR_sim_ and average SSR_Janus_ (refer back to orange dots in Figure 5 and the upper bound of the shaded region in Figure 1), alongside representative monolayers from the CBMC simulations spanning the entire range of *x*_PEG_ = 0.10 – 0.90. As *x*_PEG_ increases, we find that the Janus character of the morphologies increases approximately linearly, as highlighted in Figure 6 by the solid line that was added to aid viewing. This trend is a direct reflection of the right-skew present in the SSR_sim_ distribution shown in Figures 1 and 5. At intermediate *x*_PEG_ values (0.4 – 0.6), the DDT ligands cannot cluster onto a single facet, and the high curvature of the NP seems to promote the formation of multiple distinct DDT clusters (≈2 to 3). As *x*_PEG_ increases, the number of facets the DDT ligands cluster on is reduced, up until *x*_PEG_ = 0.9, where the DDT appears to localize entirely on one facet.

To gain additional quantitative insight about the monolayer morphology, we quantified the patchiness of the NP monolayers by measuring the ligand patch sizes for both DDT and PEG within each monolayer for all of our simulated structures. To do this, we determined the number-weighted average patch size, expressed as the number of ligands, for both ligand-types, as a function of the PEG surface fraction. The number weighted average patch size, *S*, was computed following $S=\frac{\Sigma_{i}N_{i}S_{i}}{\Sigma_{i}N_{i}}$ , where *N*_*i*_ is the number of ligands found in patch *i*, and *S*_*i*_ is the size of the patch also measured in the number of ligands such that *S*_*i*_ = *N*_*i*_ as was done in our previous publications.^6,31^ Patches were determined by identifying neighboring ligands of the same ligand-type with sulfur anchor atoms positioned within the nearest neighbor distance of 6 Å.

The results in Figure 7 reveal a direct proportionality between the patch sizes of the PEG and DDT domains and their ligand ratios, agreeing with the trend in Janus-like character reported in Figure 6. The lines in Figure 7 represent the total number of DDT and PEG ligands at each surface fraction and thus the largest possible patch size. In Figure 6, the simulation snapshot at *x*_PEG_ = 0.10 shows PEG ligands grouped between clusters of DDT, which crystallize on the flat facets of the gold NP. This morphology appears more mixed than the morphology obtained at the other extreme, *x*_PEG_ = 0.9, where the DDT all crystallize together, typically onto one facet. This difference is also supported by the SSR_sim_ values of 0.063 and 0.083 at *x*_PEG_ = 0.10 and *x*_PEG_ = 0.90, respectively. As a result, the median/maximum of the DDT cluster boxplot in Figure 7 for *x*_PEG_ = 0.90 corresponds directly to the total number of DDT ligands (*L*_DDT_ = 15) present in the simulation trials. At intermediate PEG surface fractions (*x*_PEG_ = 0.20 – 0.80), the respective patch sizes of PEG and DDT in Figure 7 change in proportion to their ligand ratios, again with DDT preferentially crystallizing on flat NP facets, as shown in Figure 6. Here, the rise in SSR_sim_ values at intermediate *x*_PEG_ is primarily due to the increased interfacial area between different ligand domains when they are both present in sizable quantities. The increased interface for phase separated systems such as these result in a more significant deviation from the corresponding binomial and thus higher SSR values. In sum, the formation of highly patchy to Janus-like PEG-DDT NP monolayers is supported by the visual representations of the computational monolayer snapshots in Figure 6 as well as the quantification of their patch sizes in Figure 7. The simulation results are confirmed by their excellent match to the experimental MALDI-MS data in Figures 4 and 5.

**Figure 7:**
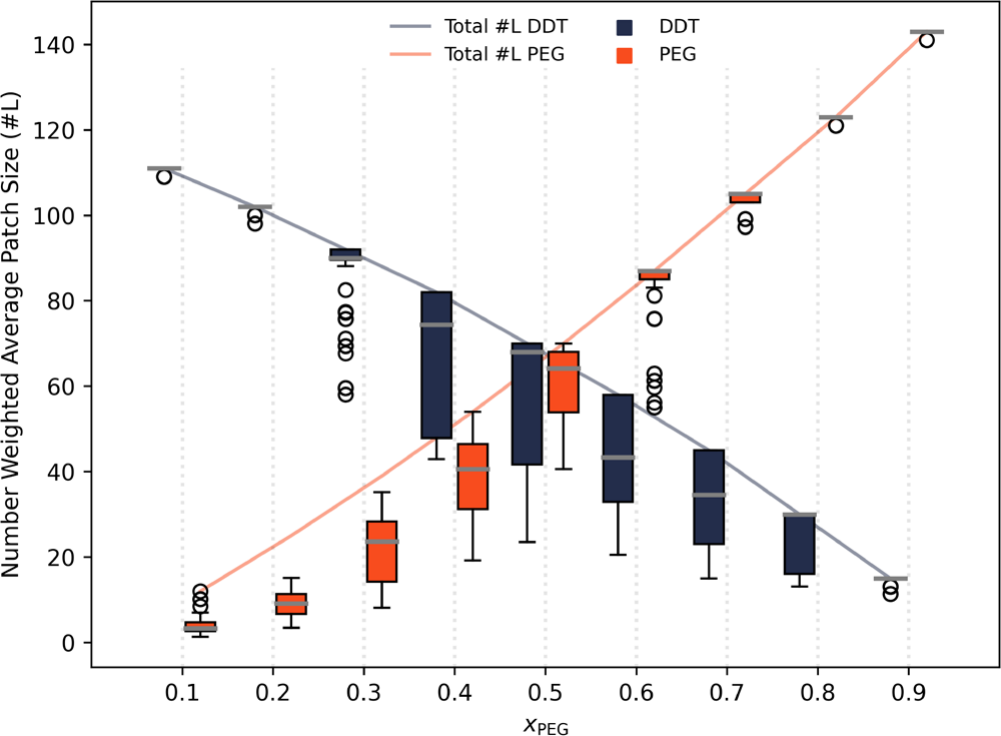
Number-weighted average patch size for both DDT (navy) and PEG (orange) patches as a function of the PEG surface fraction (indicated by the vertical dashed line that the corresponding boxes are closest to). The solid lines represent the total number of the corresponding ligand type (DDT - navy; PEG - orange) included in the simulation trials at each *x*_PEG_. Patch sizes were computed from the final 10 CBMC equilibrium monolayer configurations of each of the 18 trials per surface fraction (180 configurations were considered at each *x*_PEG_). The number-weighted average patch sizes of PEG and DDT are reported as box-plots with the gray line within each box being the median, the bounds of each box report the first and third quartile, and the whiskers indicate the minimum and maximum patch size averages within 1.5x of the interquartile range (IQR). Number-weighted patch sizes outside 1.5·IQR are reported as outliers (open circles). The number-weighted average patch sizes are also reported as the covered fraction of the NP surface area in Figure S1 of the Supporting Information.

Several physical forces are at play in the emergence of the observed patchy monolayers. First, the formation of patchy domains is driven in part by the clustering of the hydrophobic DDT ligands, facilitated by the crystallization of these longer alkanethiol chains. Prior molecular dynamics simulations on both spherical and planar gold surfaces have demonstrated crystallization behavior among alkanethiols with chain lengths exceeding 8 methylene units with the alkyl chain packing driven primarily by the surface dispersion interactions,^6,31,39,70^ consistent with the structures shown in Figure 6. Second, strong dipole–dipole interactions between the hydrophilic PEG ligands, due to the polar interactions between the partially negative ether oxygen and any neighboring partially positive PEG −CH_2_ units, contribute a significant enthalpic driving force for PEG clustering within the nanoparticle monolayer. Third, because there is also a carbon chain length difference of Δ*C* ≈ 7 between the two ligand types, there is an entropic incentive to drive the monolayer toward random or striped phases such that the conformational freedom and free volume of the longer DDT ligands are maximized. The resulting morphologies, as illustrated in Figure 6, reflect the complex interplay between entropic and enthalpic effects, giving rise to a continuum of structures ranging from sharply defined Janus configurations to more irregular patch-like domains. Importantly, the degree of Janus character increases linearly with *x*_PEG_, suggesting a tunable pathway for modulating domain morphology. This trend allows for greater control over the Janus-like character of the monolayer through the manipulation of *x*_PEG_, where Janus character increases as *x*_PEG_ approaches unity. The ability to reproducibly tune Janus character via ligand composition offers a valuable design principle for tailoring interfacial properties in nanoparticle-based systems. These findings indicate that while entropy promotes mixing of the two ligand types, the dominant enthalpic contributions—namely DDT crystallization and PEG dipole interactions—drive the system toward phase separation. In our simulations, the longer DDT ligands crystallize on flat facets of the Au NP, whereas the short PEG ligands cluster in the remaining space available on the Au NP surface prioritizing facets and edges over surface area near vertices. This preferential spatial organization enhances the Janus-like localization of the two ligands as *x*_PEG_ increases and the size of the DDT domain becomes increasingly small.

## Conclusions

We extended the integrated framework introduced in our previous publications which combines MALDI-MS experiments with CBMC simulations to investigate the self-assembly of hydrophobic dodecanethiol (DDT) and hydrophilic 2-ethoxyethane-1-thiol (PEG) ligands in ultrasmall Au NP monolayers. This framework has been shown to accurately quantify and identify key trends in monolayer morphology that provide a foundation for the theory-guided design of nanomaterials.^6,31^ Direct quantitative comparisons between the experimental and simulated MALDI-MS spectra revealed good agreement throughout the entire range of PEG-DDT surface ratios in the study. This agreement gives us additional confidence in the computational results of the PEG-DDT monolayers, which show patchy to Janus monolayer morphologies. The analysis of the simulated monolayer structures yield proportional relationships between the PEG-DDT surface ratios and their patch sizes. Hence, our finding demonstrates the ability to tune patch sizes in amphiphilic monolayers by controlling the surface ratio of the two ligand species. Our work represents a critical step forward in the synthesis and design of NPs with adsorbed amphiphilic monolayers possessing well-defined hydrophobic and hydrophilic ligand domains. Such advances open the door to engineering PEGylated NPs that could improve the performance of biocompatible sensors and enable drug-delivery applications.

## Supplementary Material



## Data Availability

The data presented in this study, along with analysis scripts, are openly available on GitHub at https://github.com/dubayresearchgroup/2025_CBMC_PEGDDT.

## Notes

SSR_exp‑sim_ values, number-weighted average patch sizes in terms of the covered fraction of the nanoparticle surface area, and additional details pertaining to the synthesis (PDF)
