# Supplementary material for: Engineering Nanoparticle Surface Amphiphilicity: An Integrated Computational and Laser Desorption Ionization Study of Controlled Ligand Self-Assembly
Source: J Phys Chem C Nanomater Interfaces. 2025 Aug 13;129(33):15097–108. doi: 10.1021/acs.jpcc.5c03644 (PMC12376114; doi:10.1021/acs.jpcc.5c03644)
Supplement: Supplementary file 1 [file jp5c03644_si_001.pdf]

# Supporting Information: Engineering Nanoparticle Surface Amphiphilicity: An Integrated Computational and Laser Desorption Ionization Study of Controlled Ligand Self-Assembly

Jacob Kennedy,[†,§] Zachary LaFaver,[‡,§] Marcus Dupart Jr.,[†] Kateri H. DuBay,[*,‡] and David L. Green[*,¶,†]

†*Department of Chemical Engineering, University of Virginia, Charlottesville, VA 22903, USA*

‡*Department of Chemistry, University of Virginia, Charlottesville, VA 22903, USA*

¶*Department of Materials Science and Engineering, University of Virginia, Charlottesville, VA 22903, USA*

§*These authors contributed equally to this work*

E-mail: dubay@virginia.edu; dlg9s@virginia.edu

# Comparison between Experiment and Simulation by means of $\mathrm{SSR}_{\mathrm{exp-sim}}$ Values

**Table S1.** $\mathrm{SSR}_{\mathrm{exp-sim}}$ values per surface fraction. The SSR values were computed using the average simulation fragment distribution compared to the nearest experimental data point's fragment distribution. Experimental data points were found to be within $\pm 0.015$ $x_{\mathrm{PEG}}$ for $x_{\mathrm{PEG}}^{\mathrm{sim}}$=0.1,0.2, and 0.8 with 3, 4, and 2 contributing experimental fragment distributions, respectively. Their average distribution, shown with standard deviation represented in the error bars (gray) in Figure 3 of the main text, was used to compute the $\mathrm{SSR}_{\mathrm{exp-sim}}$ values shown in the table below.

| $x_{\mathrm{PEG}}$ | $\mathrm{SSR}_{\mathrm{exp-sim}}$ |
|---|---|
| 0.1 | 0.0099 |
| 0.2 | 0.0049 |
| 0.3 | 0.0281 |
| 0.4 | 0.0078 |
| 0.5 | 0.0166 |
| 0.6 | 0.0190 |
| 0.7 | 0.0188 |
| 0.8 | 0.0187 |
| 0.9 | 0.0030 |

# Number-Weighted Average Patch Size Surface Fraction

To estimate the patch size measured in the number of ligands that compose a given patch to the fraction of the nanoparticle surface (consumed by the patches) we employed the following method:

1. For each configuration, the sulfur anchor coordinates were stored.

2. A KDTree was instantiated with the Au nanoparticle surface coordinates and the sulfur anchors for each cluster were iterated through. Each sulfur anchor coordinate was used to query the KDTree for all Au nanoparticle atoms within 2.6 Å. This distance threshold was determined via the Au-S radial distribution function and further confirmed by manually checking the distances between sulfurs and their surrounding Au

atoms. This distance ensures that all adjacent Au surface atoms (and only the top surface layer of Au atoms) are included as part of the corresponding cluster. This leaves us with the number of surface Au atoms covered by each cluster. Each Au atom was tracked to ensure that it was only included as part of 1 cluster.

3. Then, since we know the number of total Au atoms on the surface, we can compute the fraction of the nanoparticle surface that was consumed by each cluster, thereby giving us an estimate of the patch size in terms of the surface fraction. The total surface area of the nanoparticle utilized in this study is 25.95 $nm^2$. Thus, multiplying it by the respective median patch size surface fraction (for DDT or PEG) yields the anticipated surface area (in $nm^2$) for the DDT and PEG patch on the nanoparticle surface. The number-weighted average results are reported below in Figure S1 as box plots.

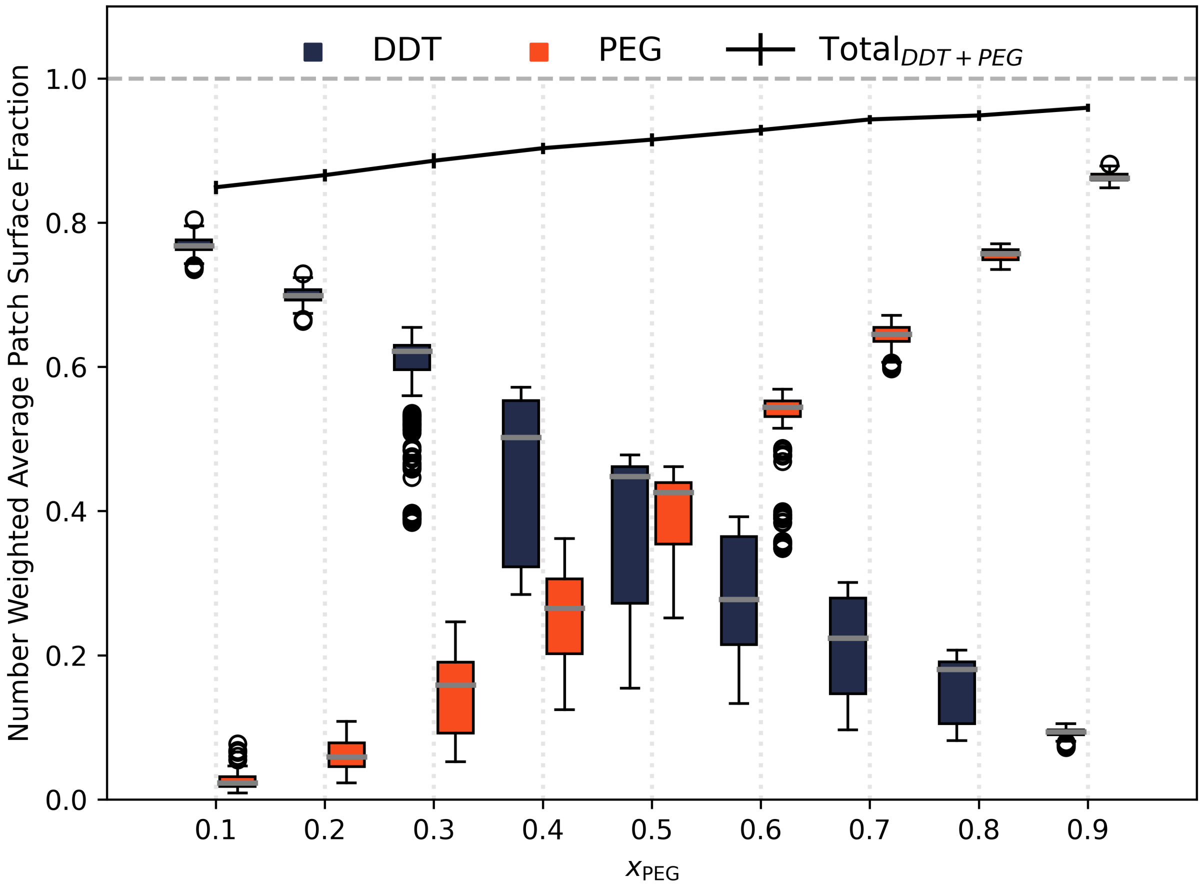


**Figure S1.** The number-weighted average patch size for both DDT (navy) and PEG (orange) patches as a function of the PEG surface fraction (indicated by the vertical dashed line that the corresponding boxes are closest to). Patch sizes were computed from the final 10 CBMC equilibrated monolayer configurations of each of the 18 trials per surface fraction (180 configurations were considered at each $x_{\text{PEG}}$). The number-weighted average patch sizes of PEG and DDT are reported as box-plots with the gray line within each box being the median, the bounds of each box report the first and third quartile, and the whiskers indicate the minimum and maximum patch size averages within 1.5x of the interquartile range (IQR). Number-weighted patch sizes outside 1.5·IQR are reported as outliers (open circles). The black line reports the total nanoparticle surface fraction consumed by both DDT patches and PEG patches with standard deviation reported in the error bars.

## Additional Experimental Data

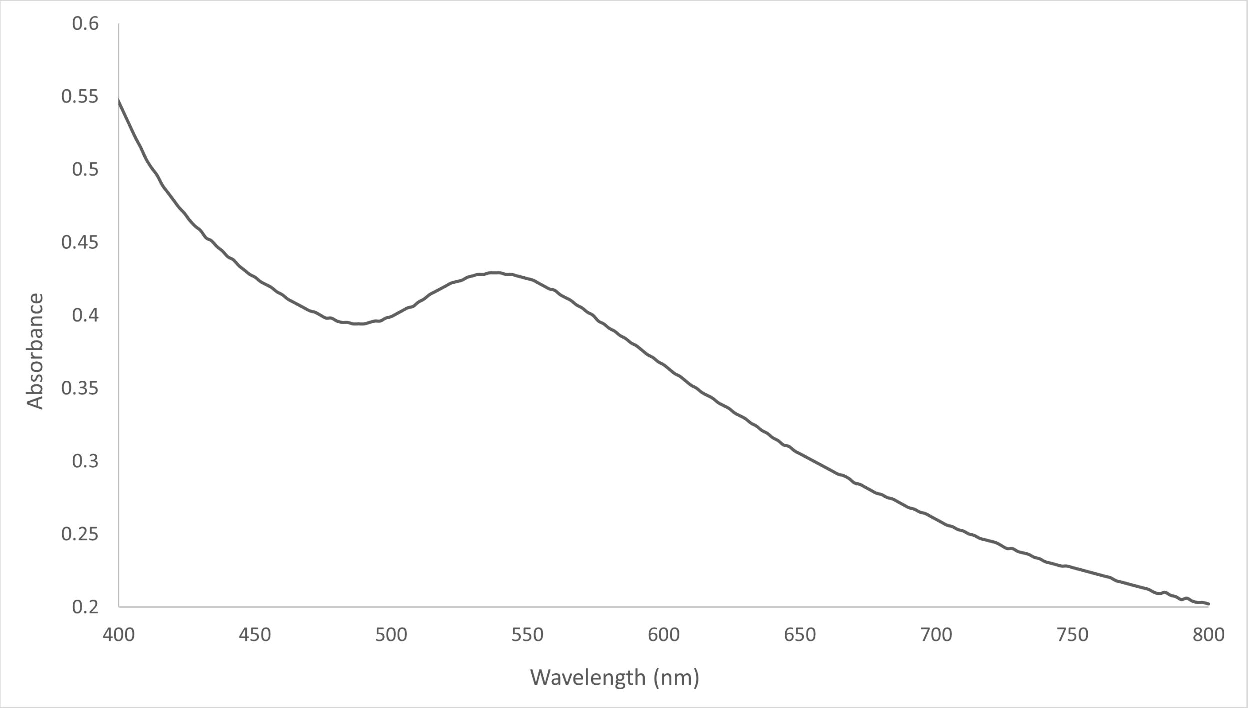


**Figure S2.** Representative UV–Vis absorption spectrum of the synthesized, monolayer-protected gold nanoparticles (AuNPs), showing a characteristic surface plasmon resonance (SPR) peak centered around 540 nm, indicative of AuNP formation.

**Table S2.** Volumes of PEG added during ligand exchange reactions (LER) and corresponding final PEG concentrations in 20 mL reaction system.

| Volume PEG Added ($\mu$L) | PEG LER Concentration (mM) |
|---|---|
| 3.76 | 0.188 |
| 4.25 | 0.213 |
| 5.40 | 0.270 |
| 7.24 | 0.362 |
| 9.30 | 0.465 |
| 14.7 | 0.733 |
| 25.0 | 1.25 |
| 58.6 | 2.93 |
| 76.1 | 3.81 |
